# A preliminary fMRI study of analgesic treatment in chronic back pain and knee osteoarthritis

**DOI:** 10.1186/1744-8069-4-47

**Published:** 2008-10-25

**Authors:** Marwan N Baliki, Paul Y Geha, Rami Jabakhanji, Norm Harden, Thomas J Schnitzer, A Vania Apkarian

**Affiliations:** 1 Department of Physiology, Northwestern University, Feinberg School of Medicine, 303 East Chicago Ave, Chicago IL, 60611, USA; 2 Rehabilitation Institute of Chicago, Northwestern University, Feinberg School of Medicine, 303 East Chicago Ave, Chicago IL, 60611, USA; 3 Departments of Anesthesia, Surgery, and Lurie Cancer Center, Northwestern University, Feinberg School of Medicine, 303 East Chicago Ave, Chicago IL, 60611, USA

## Abstract

The effects of an analgesic treatment (lidocaine patches) on brain activity in chronic low back pain (CBP) and in knee osteoarthritis (OA) were investigated using serial fMRI (contrasting fMRI between before and after two weeks of treatment). Prior to treatment brain activity was distinct between the two groups: CBP spontaneous pain was associated mainly with activity in medial prefrontal cortex, while OA painful mechanical knee stimulation was associated with bilateral activity in the thalamus, secondary somatosensory, insular, and cingulate cortices, and unilateral activity in the putamen and amygdala. After 5% lidocaine patches were applied to the painful body part for two weeks, CBP patients exhibited a significant decrease in clinical pain measures, while in OA clinical questionnaire based outcomes showed no treatment effect but stimulus evoked pain showed a borderline decrease. The lidocaine treatment resulted in significantly decreased brain activity in both patient groups with distinct brain regions responding in each group, and sub-regions within these areas were correlated with pain ratings specifically for each group (medial prefrontal cortex in CBP and thalamus in OA). We conclude that the two chronic pain conditions involve distinct brain regions, with OA pain engaging many brain regions commonly observed in acute pain. Moreover, lidocaine patch treatment modulates distinct brain circuitry in each condition, yet in OA we observe divergent results with fMRI and with questionnaire based instruments.

## Background

Mechanisms underlying CBP and OA pain states are undoubtedly different, and for the most part remain uncharacterized. Peripherally, CBP is mostly a consequence of musculoskeletal and neural damage [1], though accumulating evidence also implicates central, particularly supraspinal, processes in the signs and symptoms of the condition [2-5]. OA pain, in contrast, has generally been characterized as a chronic inflammatory response [6], although other mechanisms, such as the upregulation of Na channels [7] and increased local production of NO (contributing to cartilage degradation and inflammation) [8] have recently been identified. While there is little evidence specifically concerning central processes in OA pain, animal model studies [9,10] and human sensory testing [11] indicate reduced pain thresholds at sites removed from the OA joint that resolve in humans after joint replacement, implying that central sensitization [12] may contribute to OA pain.

A recent study characterizing pain properties on a questionnaire shows that OA and CBP (without radiculopathy) patients have similar subjective symptoms but differ from patients with signs of neuropathic pain [13]. Moreover, recent open-labeled clinical trials show the efficacy of 5% lidocaine patch (Lidoderm) therapy on pain in both CBP [14-16] and OA patients [17,18]. Lidocaine patch treatment is approved therapy for post-herpetic neuralgia (PHN) [19], and in two recent studies we described its effects on brain activity in PHN patients regarding spontaneous pain [20] and tactile allodynia pain [21], showing that spontaneous pain and related brain activity decrease with treatment, while stimulus evoked tactile allodynia and its related activity remain unaffected. Here we study brain activity for spontaneous pain in CBP and knee joint stimulation evoked pain in OA patients, comparing the effects of lidocaine therapy within and between the conditions. As CBP is manifested primarily as a continuous, spontaneous pain condition [22] and OA as more of an evoked pain state exacerbated by knee bending during sitting, walking or climbing steps [23,24], our primary hypothesis is that brain activity should be distinct between the two pain conditions. Moreover, given that in PHN patients we have shown that lidocaine therapy is more efficacious on spontaneous pain in contrast to touch-evoked pain [21,25], here we hypothesize that lidocaine therapy should be more effective on spontaneous pain in CBP patients.

## Methods

### Subjects and screening procedures

Eleven CBP and eight OA patients were recruited. Complete fMRI data were collected only in eight CBP and five OA patients and only these data were analyzed. CBP patients were included if they fulfilled the IASP criteria for CBP [26], and were diagnosed in accordance with recent guidelines [27]. OA patients were included if they fulfilled the criteria of the American College of Rheumatology for the classification of OA [28]. All the patients reported chronic pain for a duration longer than 3 months with a pain magnitude of at least 30/100 on a visual analog scale (VAS). Clinical properties are summarized in Table 1 and Table 2 respectively. Participants signed written informed consent to the experimental procedure, which was approved by the Northwestern University Institutional Review Board.

**Table 1:** Clinical characteristics for chronic back pain patients

**CBP Patients**	**Age (years)**	**Sex**	**Pain duration (years)**	**VAS**	**Below knee radiation**	**Herniated** **disc**	**Medications**
1	58	M	5	70	Yes	Yes	Ibuprofen/acetaminophen
2	29	F	3	60	Yes	No MRI	none
3	32	F	7	80	No	Yes	Valium, skelaxin, naproxen, ibuprofen
4	43	F	12	50	No	Yes	Acetaminophen/naproxen
5	59	M	30	65	Yes	No	Ibuprofen
6	47	M	10	55	Yes	Yes	Ibuprofen/Flexeril
7	68	M	4	60	No	No	Ibuprofen

Herniated disc, based on spinal MRI. M = male, F = female. VAS score is for spontaneous pain.

**Table 2:** Clinical characteristics for osteoarthritis patients

**OA Patients**	**Age (years)**	**Sex**	**Pain Duration (years)**	**VAS**	**Knee pain**	**History of trauma**	**Medications**
1	54	F	3	50	L>R	No	Celecoxib
2	61	M	30	40	R	Yes	Aspirin
3	61	M	3	60	L>R	No	Acetaminophen
4	54	M	21	80	R>L	Yes	Relafen/Aspirin
5	63	M	2.5	70	L>R	No	Ibuprofen

L = left, R = right. M = male, F = female. VAS score is for pain in the absence of stimulation.

Pain characteristics were determined using validated questionnaires. At every visit, CBP and OA patients filled the short form of McGill Pain Questionnaire (sf-MPQ) [29]. Additionally, CBP patients filled the Neuropathic Pain Scale (NPS) [30], and OA patients filled the Western Ontario and McMaster Osteoarthritis Index (WOMAC) [31].

### Experimental design and pain ratings

**Figure 1:**
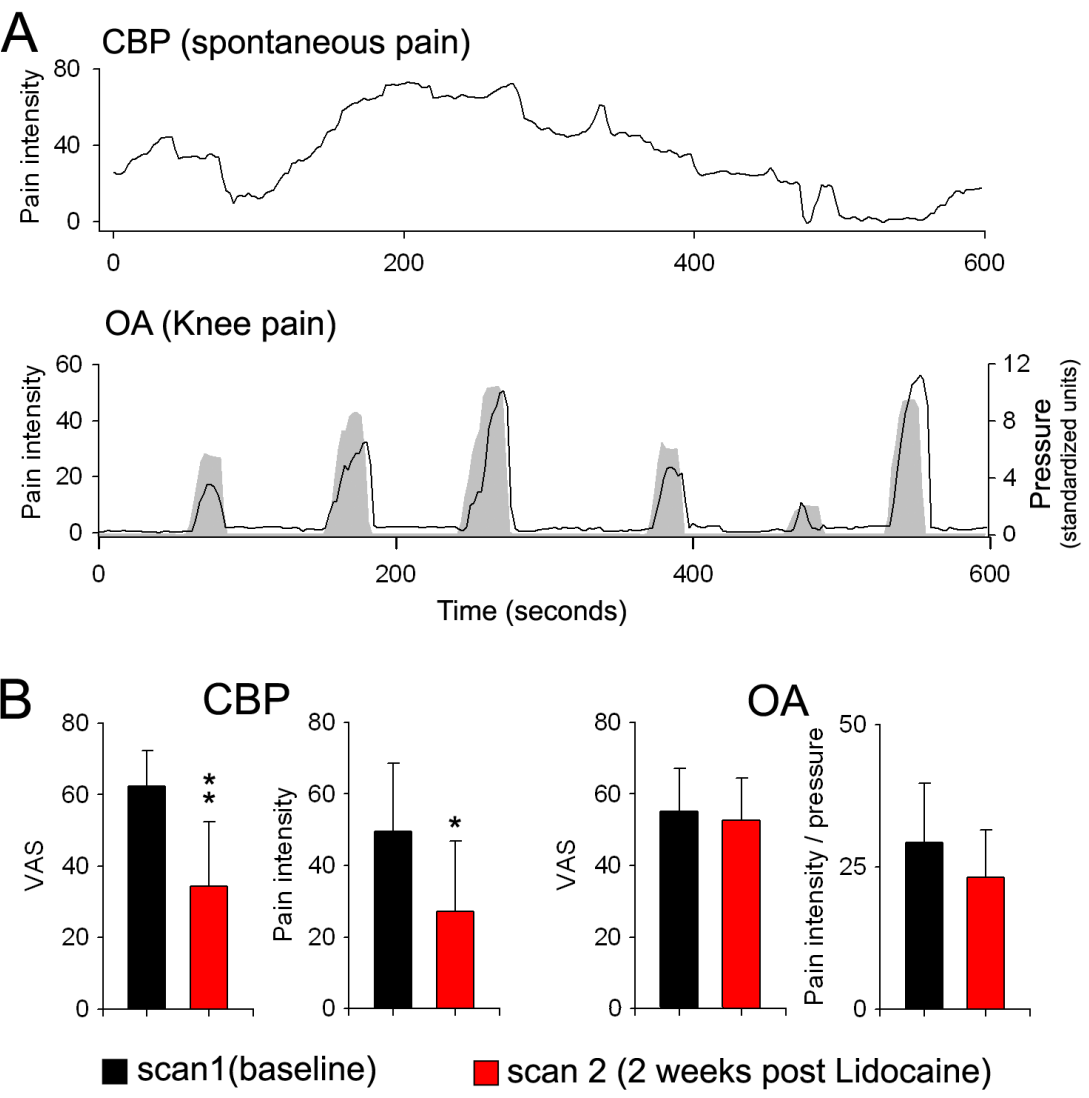
**Pain ratings and treatment effects on pain**. A. An example of pain ratings in CBP and OA. CBP patients rated spontaneous fluctuations in their pain intensity in the absence of an overt experimental stimulus (top). OA patients rated the fluctuations in their pain intensity in response to a pressure stimulus (gray) applied on the affected knee. B. CBP patients exhibited a significant decrease in pain after 2 weeks of topical Lidocaine treatment as measured by VAS (p < 0.01) and average pain during the scan (p < 0.05) (right bar graphs). OA patients did not show any decrease in pain after treatment as measured by VAS (p = 0.5), and a borderline decrease in pain in response to pressure stimulation (computed as average pain during scan divided by average pressure in standardized units) (p = 0.06) (left bar graphs). Error bars are standard deviations.

During each session patients were scanned under two different conditions: 1) pain rating (up to three scans per session) where patients were asked to report the intensity of their pain using a finger-span logging device. Prior to scanning, patients learned how to use the device to continuously report changes in their pain intensity while observing their rating projected on a computer screen as a moving bar (size of the bar is a scale from 0 to 100; 0 = no pain; 100 = worst imaginable pain); 2) one visual control scan where patients used the finger-span device to rate the length of a bar moving at a rate approximating the variability of their pain (for more details see, [32,33]. The visual task served as a motor-attention-cognitive control for non-specific brain activations due to finger motion and attention. During the pain rating task CBP patients reported the fluctuations of their spontaneous pain intensity in the absence of an overt experimental stimulus. On the other hand, OA patients rated pain in response to pressure applied to the most sensitive part of their bad knee. Pressure was delivered using a custom-made fMRI compatible device equipped with a feedback signal regarding applied pressure. The finger device and pressure signal were synchronized and time locked with the fMRI image acquisition sequence. During each scan, OA patients were presented with up to six stimulation episodes with variable intensities (range: 2–13 s.u., mean ± standard deviation : 5.5 ± 4.8 s.u., s.u. = standardized units; absolute pressure values were not used due to their strong dependence on the physical location of the stimulus on the knee), durations (range: 20 – 45 seconds, mean: 32.2 ± 15 seconds) and inter-stimulus intervals (range: 30 – 60 seconds, mean 42.8 ± 16 seconds). Examples of one CBP and one OA pain-rating task are shown in Figure 1. The applied pressure and the patient's rating of related pain were collected during scanning and used to relate brain activity to perceived pain.

### Functional magnetic resonance data acquisition

Functional MRI data was acquired with a 3T Siemens Trio whole-body scanner with echo-planar imaging (EPI) capability using the standard radio-frequency head coil. Multi-slice T2*-weighted echo-planar images were obtained with the following parameters: repetition time TR = 2.5 s, echo time TE = 30 ms, flip angle = 90°, slice thickness = 3 mm, and in-plane resolution = 3.475 × 3.475 mm^2^. The 36 slices covered the whole brain from cerebellum to vertex. Two hundred and forty volumes were acquired per scan. A T1-weighted anatomical MRI image was also acquired for each subject using the following parameters: TR = 2.1 s, TE = 4.38 ms, flip angle = 8°, FOV = 220 mm, slice thickness = 1 mm, in-plane resolution = 0.86 × 0.86 mm^2^, and number of sagittal slices = 160.

### fMRI data analysis

Image analysis to reveal significant brain activity based on changes in BOLD signal was performed on each subject's data using FMRIB Expert Analysis Tool (FEAT, [34], ). The preprocessing of each subject's time-series of fMRI volumes encompassed: slice time correction; motion correction using MCFLIRT; spatial smoothing using a Gaussian kernel of full-width-half-maximum 5 mm; nonlinear high-pass temporal filtering (Gaussian-weighted least-squares straight line fitting, filter cutoff of 100 seconds) and subtraction of the mean of each voxel time-course from that time-course (i.e. intensity normalization). The fMRI signal was then linearly modeled on a voxel by voxel basis using FMRIB's Improved Linear Model (FILM) with local autocorrelation correction [35,36].

Each condition described above (rating of spontaneous pain in CBP, rating of stimulus pain in OA, and rating of visual stimuli in both populations) was considered to generate a hemodynamic response described by the convolution of the corresponding vector with a canonical hemodynamic response function (gamma function: lag = 6 seconds, standard deviation = 3 seconds). Head motion vector (derived from motion correction) was used at this level as a covariate of no interest to further remove any residual variance due to head motion. The significance of the model fit to each voxel time-series was calculated, yielding statistical parametric maps for the pain and visual rating conditions in each subject.

**Figure 2:**
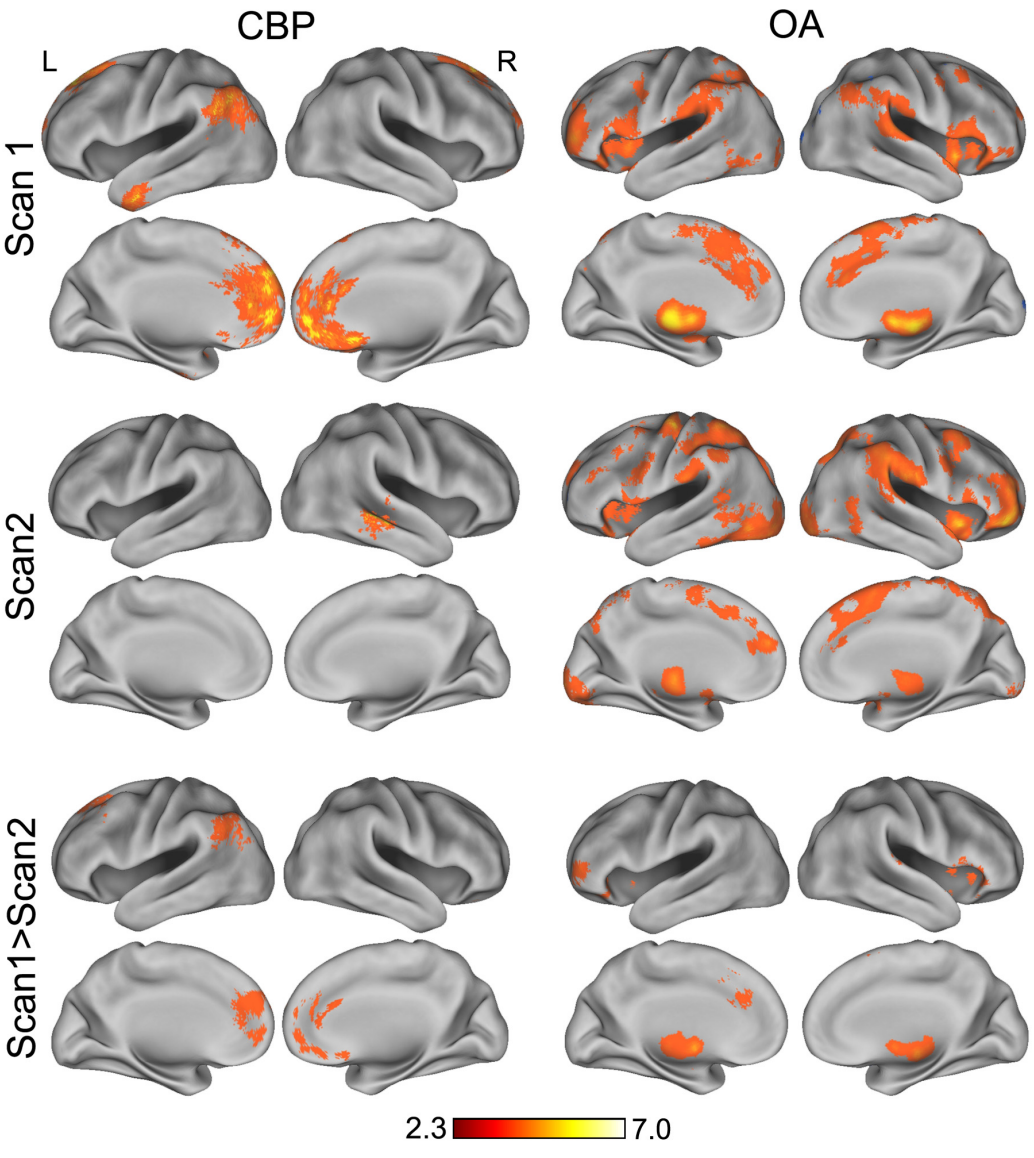
**Brain activity maps for CBP and OA**. CBP and OA group average brain activity maps (fixed effects analysis) for session1 (prior to treatment), session 2 (2 weeks after application of Lidoderm) and contrast between before and after treatment (scan 1 > scan 2). Each scan map is a subtraction between the pain rating task and the control visual rating task.

Average group activity pain maps for OA and CBP for in session 1 and session 2 were generated by first subtracting the visual task related activity from the pain related activity map for each subject using FEAT in a higher level fixed effects analysis following the co-registration of individual scans to standard space (152 subject average MNI space, ). This results in a contrast of parametric estimate (COPE) map of statistically significant pain minus visual activity for each patient. Group average pain minus visual activity maps were then generated by averaging COPE maps for CBP (n = 7) and OA (n = 5) in a third higher level fixed effects analysis. The resultant group average pain minus visual maps were thresholded at z-scores > 2.3, and a cluster probability threshold of p = 0.01 was applied to compute the significance of each cluster, thereby correcting for multiple comparisons [37]. Group average pain minus visual brain activity for CBP and OA are shown in Figure 2.

Brain regions exhibiting a significant decrease in activity in response to lidoderm treatment in CBP and OA were identified using a paired t-test (fixed effects analysis, z-score > 2.3, cluster threshold p = 0.01) between the higher-level (pain – visual) COPEs statistical images of session 1 and session 2. The resultant z-map for session 1 minus session 2 for each patient population was then masked with its respective group average activity of the pain minus visual of session 1 to eliminate false positive results. These maps thus depict the brain areas showing pain-specific activity that significantly decreased with treatment.

**Figure 3:**
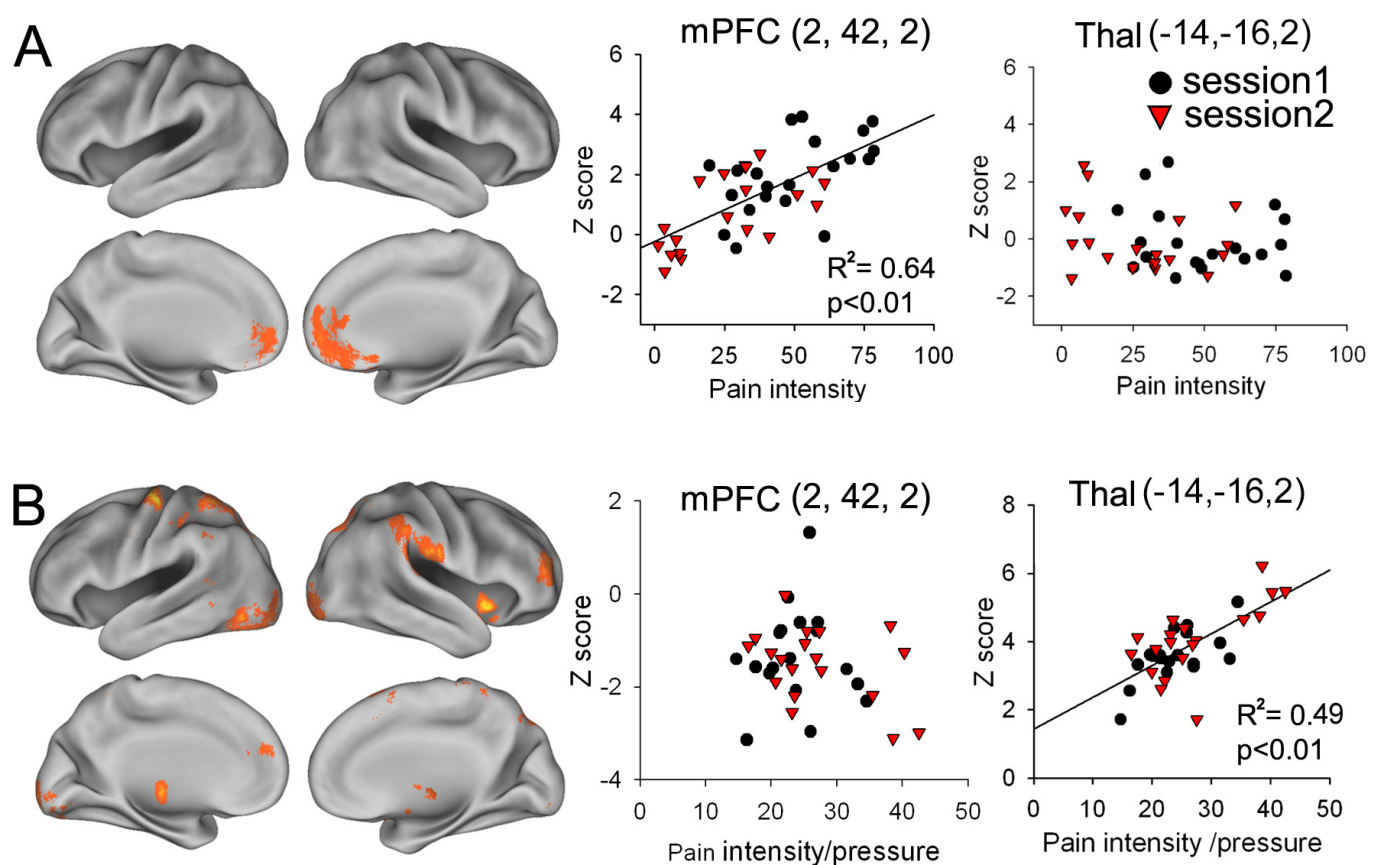
**Covariate analysis of brain activity with pain intensity in CBP and OA**. A. Covariate map shows brain regions that encoded pain intensity in CBP. It identifies the rostral ACC and mPFC. Scatter plots show the relationship between pain intensity and z-values (averaged within 3 × 3 cluster around the peak) in mPFC and right thalamus (identified from OA covariate analysis) in CBP. B. Covariate map for OA. It shows many brain regions, none overlap with the region seen in CBP. Scatter plots are in OA for the same regions shown in A.

To determine those brain regions encoding pain intensity, we performed a whole brain covariate analysis using FSL (fixed effects analysis, z-score > 2.3, cluster threshold p = 0.01) across CBP and OA patients. For CBP, the first level pain activity maps were pooled from session 1 and 2 (n = 42) and regressed with the average spontaneous pain during the scan. Similarly, the first level OA pain activity maps were pooled from sessions 1 and 2 (n = 37) and regressed with average pain divided by applied pressure, thereby normalizing perception to unit intensity of applied stimulus. We used standardized stimulus units rather than absolute values of pressure to account for the variability of pressure intensities necessary to induce pain across the knee. The z-maps for the covariate analysis for CBP and OA were then masked with their respective group average activity for pain minus visual contrast, to restrict the analysis to pain specific brain regions. To show the relationship of brain activity and pain intensity in more detail, we also performed a region of interest analysis (ROI). For each scan, we extracted the z-values of a 1 cc ROI centered on the peaks obtained in the covariate maps and examined their relationship to perceived pain (Figure 3).

To delineate the brain regions encoding pain intensity that were also modulated by treatment, we performed a whole brain voxel-wise conjunction analysis between the thresholded z-map for treatment and the pain intensity covariate maps for CBP and OA. For each patient group we then identified the ROIs from the conjunction map and extracted their z-values from a 1 cc volume and used them to perform an unpaired t-test between session 1 and session 2.

### Surface-based mapping

Surface-based mapping (figures 2 and 3) was constructed using the Population-Average, Landmark- and Surface-based (PALS) average fiducial surface from 12 individual subjects as the atlas target [38].

## Results

### Behavior

CBP patients showed a robust decrease in pain intensity as well as other clinical pain parameters in response to a two-week topical Lidocaine treatment applied to the back. Pain intensity was assessed from VAS scores obtained in sf-MPQ, and by averaging the spontaneous pain reported by the subject during the scan. Both of these measurements exhibited significant reduction after treatment (Figure 1B, Table 3). In addition, CBP patients showed a significant decrease in the surface score (p < 0.05) and borderline significant decreases for the deep, unpleasantness, and overall neuropathic pain score (Table 3).

**Table 3:** Pain parameters and clinical data for CBP and OA patients

**CBP**				**OA**			
	**Session1**	**Session2**	**p-value**		**Session 1**	**Session 2**	**p-value**
Pain Int.	49.5 ± 19	27.2 ± 18.1	0.034	Pain Int.	39.6 ± 14.3	37.2 ± 12.6	0.513
VAS	62.3 ± 9.9	34.3 ± 19.6	0.008	VAS	55.5 ± 12.3	52.5 ± 11.9	0.501
Affective	3.4 ± 4.5	2.3 ± 4.4	0.331	Affective	0.5 ± 1.0	1.5 ± 1.0	0.308
Sensory	11.1 ± 9.2	8.3 ± 10.1	0.482	Sensory	9.3 ± 1.7	6.3 ± 3.9	0.334
NPS (*N*)	17.8 ± 13.7	9.9 ± 8.4	0.055	Pain (*W*)	10.0 ± 1.6	9 ± 2.4	0.252
Unpleas. (*N*)	5.2 ± 1.3	3.4 ± 2.1	0.094	Stiffness (*W*)	5.0 ± 1.4	5.0 ± 1.4	1.000
Deep (*N*)	6.6 ± 1.7	3.5 ± 2.6	0.082	Activity (*W*)	35.2 ± 4.9	31.5 ± 9.1	0.243
Surface (*N*)	4.2 ± 1.9	1.9 ± 2.3	0.025	Pain Int./Pr	29.3 ± 10.4	23.1 ± 8.3	0.0622

*Session 1 *= prior to use of Lidoderm therapy; *Session 2 *= after 2 weeks of Lidoderm therapy. *Pain Int. *= mean pain rating during the scan; *Pain Int./Pr *= mean pain rating during the scan divided by normalized applied pressure.*VAS *= visual analog scale; Unpleas = unpleasantness; *VAS, Affective and Sensory *are from McGill short form. N = Neuropathic Pain Scale; W = WOMAC. All parameters are expressed as μ ± S.D. p values were computed using a paired t-test.

In contrast to CBP, OA patients did not show improvement after Lidocaine treatment in their knee pain as measured by VAS in sf-MPQ, or by intensity of mean pain ratings for knee stimulation, and other OA related pain characteristics on either sf-MPQ or on WOMAC. They did exhibit however a borderline decrease in ratings of knee pain (p = 0.06) after normalization with applied pressure (Figure 1B, Table 3).

### Brain activity in CBP and OA

Before treatment (session 1), spontaneous pain in CBP gave rise to increased brain activity mainly in the frontal cortex, including the medial prefrontal cortex (mPFC), the rostral anterior cingulate cortex (rACC), bilateral superior frontal gyrus (SFG), and the nucleus accumbens (NAc). Increased activity was also observed in the inferior temporal gyrus (ITG), and the left posterior parietal cortex (PP). Therefore, CBP is represented mainly in emotion/reward (mPFC and NAc) mediating areas as reported previously [39]. After treatment (session 2), only a small cluster in the left MT area showed significant increased activity for spontaneous pain (Figure 2, Table 4).

**Table 4:** Brain regions activated for CBP and OA

**Brain region**	**Session 1**		**Session 2**		**Session 1 > Session 2**	
	**Coordinates** **x, y, z**	**Z-score**	**Coordinates** **x, y, z**	**Z-score**	**Coordinates** **x, y, z**	**Z-score**
***CBP (Pain-Visual)***						

mPFC (32/10)	2, 48, 2	10.5			6, 52, -8	5.85
R ACC (32)	-2, 38, 20	7.72			-2,38,20	4.31
L SFG (8)	-16, 32, 46	9.76			-16,30,44	6.91
R SFG (8)	16,22,52	7.66				
NAc	6, 12, -10	8.46				
L ITG (20)	-56, -8, -32	6.36				
L PP (7)	-48, -72, 38	8.18			-46, -74,44	6.12
L MT			-64, -44, -10	4.12		

**OA *(Pain-Visual)***						

R VLOFC (10/11)			44, 42, -14	6.27		
L MFG (9/46)	-36, 42, 6	4.88			-38, 52, 12	4.19
R MFG (9/46)			-40, 36, 32	4.78		
ACC (32)			-4, 38,22	4.96		
L Ant Insula/IFG	-36, 28, -8	4.07			-36, 28, -8	4.39
ACC (24)	-8, 22, 28	4.34	6, 30, 26	4.57	-4, 24, 28	2.63
R MFG (8/9)	50, 14, 48	3.7	40, 18, 52	4.65		
L Putamen			-22, 14, -2	4.59		
SMA (6/8)	2, 8, 62	4.25	10,16,62	4.35		
L Insula (48)	-36, 6,4	3.72	-40, 8, -6	5.12		
R Insula (48)	42, 6, -4	5.75	38, 18, -8	3.09		
R Putamen	26, 6, -2	3.23	-18, 14, -2	6.22		
L Amygdala	-20, -6, -18	3.17				
R Thalamus	14, -18, 2	5.6	16, -18, 4	4.39	12, -18, 0	4.00
L Thalamus	-8, -22, 0	6.14	-16, -16,4	4.42	-6, -22, 0	3.62
L SI/MI (3/4)			-38, -24,56	6.12		
L S2	-54, -26, 24	4.87	-58, -28, 26	5.01		
R S2	52, -28, 24	2.87	54, -24, 26	4.67		
Pre-cuneus (7)			0, -52, 68	5.88		
L PP (7)			-28, -52, 54	5.46		
L MT			-56, -64,4	5.36		
R PP			22, -76,48	5.5		
L Visual cortex			-12, -80, -12	6.1		

L = left; R = right; mPFC = medial prefrontal cortex; DLPFC = dorsolateral prefrontal cortex; S2 = secondary somatosensory cortex; M1 = primary motor cortex; ACC = anterior cingulate; SMA = supplementary motor area; LP = lateral parietal cortex; PP = posterior parietal cortex; IFG = inferior frontal gyrus; NAc = nucleus accumbuns; ITG = inferior temporal gyrus; SFG = superior frontal gyrus; MT = middle temporal cortex; VLOFC = ventrolateral orbitofrontal cortex. Numbers in parenthesis = Brodmann areas. Minus denotes left, posterior and ventral to the anterior commissure for x, y, and z mm, respectively.

Evoked knee OA pressure-pain, on the other hand, showed more extensive activity pattern, with similarities to activity for acute pain in normal subjects [40]. Before treatment (session 1), painful knee stimulation evoked activity in: bilateral thalamus, secondary somatosensory cortex (SII), insula, supplementary motor area (SMA), anterior cingulate (ACC) and medial frontal gyrus; and unilaterally in right putamen, and left amygdala. After two weeks of treatment (session 2), the brain activity map was similar with some exceptions. No significant activity was observed in the left amygdala, right putamen, and left anterior insula. Additionally a new, large activation was observed in right ventrolateral orbitofrontal (VLOFC), as well as more activations in mid temporal and visual cortices (Figure 2, Table 4).

### Treatment effect on brain activity

As we noted earlier, treatment of CBP with lidocaine led to a significant decrease in pain intensity rating. This was accompanied by a significant decrease in brain activity in mPFC, rACC, SFG, and PP when maps from session 1 and 2 were statistically contrasted. In OA, lidocaine treatment only showed a borderline decrease in pain per applied pressure, but the statistical contrast between session 1 and 2 indicated significantly decreased brain activity in left MFG, left anterior insula, and bilateral thalamus (Figure 2, Table 4).

### Covariate analysis

Brain areas encoding pain intensity in CBP and OA were identified using a covariate analysis. In CBP the covariate was mean spontaneous pain during the scan, and in OA the covariate was the mean pain during the scan divided by the normalized applied pressure. The mPFC and parts of the rACC at the level of the genu encoded pain intensity in CBP (figure 3A). In contrast, the mid ACC, right insula, bilateral thalamus, right S2 and right MFG encoded pain/pressure in OA (figure 3B). Thus, pain intensity representation in CBP and OA (normalized with pressure in OA) pain map to different brain regions.

To show the relationship of brain activity and pain intensity in more detail, we performed a region of interest analysis. For each scan, we extracted the z-values of a 1 cc ROI centered on the peaks obtained by the covariate maps. The ROIs used include mPFC (2,42,2), left thalamus (12,-20,4), right insula (40,8,-4), right S2 (46,-18,32), mid ACC (-4,24,28) and right middle frontal gyrus (MFG) (38, 38, 36). The mPFC showed a significant correlation with pain intensity for CBP (p < 0.01) but not for OA, while the left thalamus was positively correlated with pain intensity for OA (p < 0.01) but not CBP (figure 3). The insula, S2 mid-ACC, right MFG also showed positive correlations with pain intensity for OA, with p values of 0.035, 0.042, 0.041, and 0.039 respectively, but not for CBP.

### Change in brain regional activity with treatment

The covariate analysis indicates the brain regions where brain activity reflects pain intensity, while the contrast between the pain rating and visual rating tasks between session 1 and 2 shows brain regions specifically involved in pain that decrease with treatment. To identify brain regions that *both *code pain and decrease with treatment we performed a conjunction analysis between the covariate and contrast analyses, for both OA and CBP patients. In CBP this resulted in a single cluster in the mPFC (-2, 48, 2) with activity z-value of 3.11 ± 1.78 in session 1 and 0.98 ± 0.98 in session 2, showing a large treatment effect (t = 8.386; p < 0.001). In OA only bilateral thalamus survived: with the larger change seen in left thalamus (-12, -20, 2; with session 1 z-value of 3.21 ± 0.93; session 2 z-value of 1.12 ± 0.96; and treatment effect t = 8.89; p < 0.001); and a similar but smaller change seen in the right thalamus (16, -18, 4; session1 z-value 2.98 ± 0.92; session 2 z-value 0.97 ± 0.88; and treatment effect t = 7.15; p < 0.01).

## Discussion

A wealth of data exists regarding the clinical pain characteristics of CBP and OA. To our knowledge, this is the first study examining brain activity in both conditions, before and after an analgesic treatment the efficacy of which remains unproven in both conditions.

The results indicate that brain activity in CBP for spontaneous pain is different from brain activity for mechanically evoked knee pain in OA. While CBP pain related brain activity mapped mainly to the mPFC and adjacent rACC, the evoked OA pain mapped to many areas commonly seen in acute pain in [41], in addition to regions that seem more uniquely activated in the condition: the MFG, and amygdala. Moreover, the pattern of pain intensity encoding was distinct between evoked OA pain and spontaneous CBP. While the former correlated to brain activity in the thalamus, insula, mid-ACC, S2, and MFG, the latter correlated to activity in mPFC/rACC. Furthermore, modulation of afferent input with lidocaine patches gave rise to different patterns of change in behavior and in brain activity depending on the condition. The results corroborate our general hypothesis, that different clinical chronic pain conditions have distinct brain activity patterns, which in part is a function of the type of pain investigated.

Both patient groups studied use similar drugs for pain relief, and have similar durations of chronicity and clinical characteristics. As both groups also describe their pain similarly on the NPS scale [42], and as recent studies show efficacy of lidocaine patch treatment in pain relief in both groups [14,15,18,43,44], we reasoned that comparing brain activity and responses to this treatment between the two groups was meaningful. Despite these similarities, brain activity is distinct for knee stimulation pain in OA from spontaneous pain in CBP.

We examined spontaneous pain in CBP, as this is the most relevant clinical pain issue in CBP patients. On the other hand, we resorted to studying knee-stimulation evoked pain in OA because there was minimal fluctuation in spontaneous pain when these subjects were at rest and the knee not provoked. It therefore remains unclear whether OA patients even have spontaneous pain or if the non-stimulated pain reported by OA patients is a reflection of the after effects of manipulations to the knee. A recent study described brain activity in OA in the absence of stimulation [45]. Thus, their results might reflect brain regions involved in spontaneous pain in OA. The reported activation pattern resembles activity for knee-stimulation evoked pain described here, and also a large mPFC activation that matches the activity we see for spontaneous pain in CBP. Kulkarni et al. mention that the pain was a reflection of the "ongoing knee pain experienced during patients' accustomed physical activities", suggesting that the reported activity may be due to previous provocation of the painful joints. Still, it should be emphasized that Kulkarni et al.'s results highlight the fact that OA pain engages sensory regions of the cortex even in the absence of a stimulus.

This study and our previous work [46,47] demonstrate that spontaneous pain intensity encoding is comparable between CBP patients and post-herpetic neuralgia (PHN) patients. In both cases it is encoded, though with some differences, in areas normally involved in hedonic experience and emotional learning, such as the mPFC/rACC and orbitofrontal cortex [48-52], in reward and goal directed behavior such as the striatum [53], and in fear behavior such as the amygdala [54]. Furthermore, those are the primary areas modulated by lidocaine patch treatment in both conditions. Thus both CBP and PHN spontaneous pain appear to be primarily emotional states, with lidocaine patch therapy decreasing this emotional pain. In contrast, knee-stimulation evoked pain related brain activity was mostly sensory and the activity in these sensory pain representational areas specifically decreased with lidocaine patch therapy.

Lidocaine patch acts through blockade of abnormally functioning (sensitized) Nav1.7 and Nav1.8 Na+ channels in dermal nociceptors, thereby reducing ectopic discharges [55,56]. Lidocaine has also been shown to regulate T-cell activity and inhibit NO production [57,58]. It is thus likely that the decreased pain perception with lidocaine patch therapy is due to its effect on sensitized nociceptors, as well as to decreasing inflammatory processes within the deep tissue such as injured muscle, joint, tendon, and constricted nerves that provide the afferent input contributing to the pain of CBP and/or OA. The extent to which lidocaine can penetrate these injured structures in the deep tissue may be one explanation for its better effectiveness in CBP. We cannot, however, distinguish between multiple implications: distinct fiber types being involved in each type of pain and yet both responding to lidocaine patch therapy; same afferents being modulated with the therapy but distinct spinocephalad pathways signaling nociceptive inputs, and thus affecting different cortical activity; or some combination of both.

The evoked OA pain in this study elicits activity patterns and engages brain regions similar to those observed in touch-evoked pain (allodynia) [21,59-61] and acute pain [62]. However, intensity encoding of pain appears to be represented by brain activity patterns specific to each clinical condition. The insula and S2 in OA patients in the present study, and in one case of psoriatic arthritis [63], encode evoked pain intensity and these two regions were responsive to a COX2-inhibitor that resulted in pain relief in the psoriatic arthritis patient. However, in OA only bilateral thalamus encoded pain intensity and responded to lidocaine patch therapy. In comparison, in PHN patients basal ganglia and medial temporal gyrus responses were related to the ratings of tactile allodynia [21].

The present results and earlier human brain imaging evidence for differential involvement of distinct brain regions in various clinical chronic pain conditions is consistent and complimentary to accumulating evidence in animal studies regarding the differential involvement of cortical regions in various animal models of chronic pain. Recent studies indicate that neuronal activity and receptor properties of the amygdala are modified in persistent pain conditions and these changes influence spinal cord neuronal activity [64,65]. Changes in synaptic efficacy and second messenger properties have also been observed in the ACC in animal models of chronic pain, which were correlated to the pain behavioral outcomes [66,67]. Thus these studies potentially provide mechanistic explanations for the different brain activity associated with OA and CBP and their possible contribution to the resultant perceptions.

### Technical issues

If we examine pain measures either by ratings or by questionnaire outcomes in the OA group, no treatment effects are observed. The borderline treatment effect only shows up when pain is divided with applied pressure, and this parameter relates to statistically significant brain activity changes. This is a normalization of perception per unit stimulus, it is equivalent to earlier efforts of matching pain perception by varying stimulus strength [68-70], and might be a critical step in assessing pain treatment effects on evoked pain, especially in clinical pain conditions.

In the present study, while the number of participants in each group was small, the data was nonetheless reproducible enough to show significant changes in brain activity. Due to the small number of subjects studied we have used fixed effects statistics. Therefore, the obtained results cannot be generalized to the population at large, neither for CBP nor for OA.

Here we demonstrate that decreased brain activity with treatment may be identified prior to the patients' report of decreased pain on standard questionnaires commonly used in clinical trials. Open labeled clinical trials indicate decreases in OA pain, as measured by WOMAC or NPS [18,71], even in small groups of patients. Therefore, it is not clear why our OA patients did not have clinical signs of decreased pain. The design of the present study was based on the idea that painful mechanical stimuli applied to the knee are relevant to knee OA, and that manipulation of this pain should also have clinical relevance. We are surprised that even though knee stimulation evoked pain is modulated with the treatment, clinical questionnaires based outcomes are not. This is in contrast to the CBP patients, at least suggesting that lidocaine therapy is more efficacious in CBP. The discrepancy between questionnaire outcomes and stimulus-evoked pain implies that in OA the clinical pain depends on factors more than just the knee pressure-pain relationship. Therefore, although we observe that lidocaine patch therapy modulates the pressure-pain relationship and thalamic activity reflects this modulation, we cannot conclude that this effect is clinically significant. Alternatively, these results in OA suggest that perhaps continued treatment for longer durations and at higher doses may result in a clinically significant outcome.

## Conclusion

Overall, the diverse brain activation patterns in these two groups of patients indicate that brain circuitry underlying CBP spontaneous pain and OA joint stimulation evoked pain are distinct. Moreover, distinct brain regions encode and respond to lidocaine patch therapy. We also demonstrate that, even in the absence of questionnaire based evidence for pain relief; fMRI may provide clues suggesting analgesic effects of a therapy. These studies lack a placebo arm and were performed in a small number of patients. The available data and the limited number of studies in the topic, therefore, underscore the need for larger, placebo controlled fMRI trials for efficacy of lidocaine patch therapy in both CBP and OA patients.

## Competing interests

The authors declare that they have no competing interests.

## Authors' contributions

MB participated in functional data acquisition, performed the statistical and data analysis and drafted the manuscript. PG participated in data acquisition and analysis. RJ participated in experimental design of the study and data acquisition, and provided technical support. NH and GT aided in patient recruitment screening and coordination. AVA conceived the study, participated in design and statistical analysis, and is the primary author of the manuscript. All authors read and approved the final manuscript.
